# Exploration of the Immune-Related Signatures and Immune Infiltration Analysis in Melanoma

**DOI:** 10.1155/2021/4743971

**Published:** 2021-01-16

**Authors:** Ai-lan Li, Yong-mei Zhu, Lai-qiang Gao, Shu-yue Wei, Ming-tao Wang, Qiang Ma, You-you Zheng, Jian-hua Li, Qing-feng Wang

**Affiliations:** ^1^Department of Dermatology, Dongying People's Hospital, Dongying 257091, China; ^2^College of Integrated Chinese and Western Medicine, Liaoning University of traditional Chinese Medicine, Shenyang 110079, China

## Abstract

In the present study, we aimed to investigate immune-related signatures and immune infiltration in melanoma. The transcriptome profiling and clinical data of melanoma were downloaded from The Cancer Genome Atlas database, and their matched normal samples were obtained from the Genotype-Tissue Expression database. After merging the genome expression data using Perl, the limma package was used for data normalization. We screened the differentially expressed genes (DEGs) and obtained immune signatures associated with melanoma by an immune-related signature list from the InnateDB database. Univariate Cox regression analysis was used to identify potential prognostic immune genes, and LASSO analysis was used to identify the hub genes. Next, based on the results of multivariate Cox regression analysis, we constructed a risk model for melanoma. We investigated the correlation between risk score and clinical characteristics and overall survival (OS) of patients. Based on the TIMER database, the association between selected immune signatures and immune cell distribution was evaluated. Next, the Wilcoxon rank-sum test was performed using CIBERSORT, which confirmed the differential distribution of immune-infiltrating cells between different risk groups. We obtained a list of 91 differentially expressed immune-related signatures. Functional enrichment analysis indicated that these immune-related DEGs participated in several areas of immune-related crosstalk, including cytokine-cytokine receptor interactions, JAK–STAT signaling pathway, chemokine signaling pathway, and Th17 cell differentiation pathway. A risk model was established based on multivariate Cox analysis results, and Kaplan-Meier analysis was performed. The Kruskal-Wallis test suggested that a high risk score indicated a poorer OS and correlated with higher American Joint Committee on Cancer-TNM (AJCC-TNM) stages and advanced pathological stages (*P* < 0.01). Furthermore, the association between hub immune signatures and immune cell distribution was evaluated in specific tumor samples. The Wilcoxon rank-sum test was used to estimate immune infiltration density in the two groups, and results showed that the high-risk group exhibited a lower infiltration density, and the dominant immune cells included M0 macrophages (*P* = 0.023) and activated mast cells (*P* = 0.005).

## 1. Introduction

As one of the most common cutaneous malignancies in the clinic, the incidence of melanoma is dramatically increasing, with an annual rate of 75,000 cases/year, representing 4.5% of all US cancer cases [1]. In 2012, 232,000 cases of melanoma were newly diagnosed and 55,000 deaths were reported globally [2]. Malignant melanoma (MM) is highly metastatic. Around 50–80% of patients with advanced melanoma display liver metastases [3, 4], and more than one-third of advanced melanoma cases have brain metastases [5, 6]. MM has the highest mortality rate among other cutaneous malignant tumor types, and the median survival (MS) of advanced melanoma patients with metastasis is 8–12 months [7, 8]. Besides, the metastasis location strongly influences the 5-year overall survival (OS) of patients with MM: the 5-year OS of M1a, including, skin, subcutaneous tissue, and extraregional lymph node metastases, is around 23%, while that of M1b (lung metastases) is 17% [9]. Patients with visceral metastases (M1c), especially in the brain and liver, have been shown to exhibit a 5-year OS of less than 10%, with an MS of below 1 year [10].

After treatments, such as local-regional therapy, radiotherapy, and chemotherapy which have been tested to prolong OS and MS, immunization therapy has gradually become the primary means of MM treatment. The Food and Drug Administration-approved agents include interferon *α*2b (IFN-*α*2b) [11] and high doses of interleukin (IL-2, 12), while vemurafenib [12], mitogen-activated protein kinase/extracellular signal-regulated kinase kinase inhibitor [13], encorafenib [14], and binimetinib [15] are commonly used as targeted drugs against this malignancy. These data have triggered an interest in the application of immunotherapy for MM. However, further studies on the immune mechanism of MM are needed to help provide a theoretical basis for the clinical use of these agents and identify potential molecular targets for MM diagnosis and treatment.

Although several factors contribute to the occurrence and prognosis of MM, immune response signaling pathways are considered key factors and have been correlated with the development of an MM-immune microenvironment [16]. However, lack of new tumor antigens, the insufficient number of tumor-infiltrating lymphocytes (TILs) [17], and overexpression of immunosuppressive molecules, such as programmed death 1/programmed death-ligand 1 [18], wingless-type MMTV integration site family, member 5A [19], and signal transducer and activator of transcription 3 (STAT3) inhibitor [20], inhibit tumor immunology, leading to the deterioration of MM-immune microenvironment. Hence, elucidating the immune microcharacteristics of MM can effectively counteract immune evasion, thus prolonging the OS of patients with MM. Immunotherapies, such as use of sorafenib, a protooncogene serine/threonine-protein kinase (RAF) inhibitor, which targets the RAF, platelet-derived growth factor receptor, vascular endothelial growth factor receptor, and Fms-like tyrosine kinase 3, have been shown to induce caspase-independent apoptosis in melanoma cells [21]. Another study reported that, in patients with melanoma, treatment with tremelimumab, a monoclonal antibody against cytotoxic T lymphocyte antigen-4, activated positive immune responses and maintained T cell responses by excess generation of CD8^+^ T cells, thus blocking CTLA-4 [22].

In MM, the tumor immune environment, especially TILs, has been correlated with patient therapy and prognosis [23]. Hence, it is essential to conduct research on immune infiltration in MM. Previous studies mainly focused on differential gene expression in the tumor immune environment in MM, while the effect of MM immune environment on patient prognosis remains poorly understood. Moreover, information on immune signature distribution in the MM microenvironment is still lacking. In order to identify robust immune signatures that can serve as potential therapeutic targets, it is essential to further investigate the relationship between specific biomarkers of tumor immune cells and MM.

In the present study, we obtained transcriptomic data from The Cancer Genome Atlas (TCGA) and screened for immune-related genes using an immune-related signature list from the InnateDB database. To evaluate the clinical OS of MM patients with these immune-related genes, we performed correlation using a multivariate Cox regression model and constructed a risk model of MM based on its results. To further investigate the correlation between this risk model and clinical characteristics, we performed correlation analyses. Finally, we utilized CIBERSORT and TIMER databases to evaluate the association between hub immune signatures and immune cells in the MM immune microenvironment and determined the characteristics of immune-infiltrating cells in MM.

## 2. Methods and Materials

### 2.1. Data Acquisition and Processing

From TCGA database (https://portal.gdc.cancer.gov/), we downloaded the genome expression data of 472 samples, including 471 tumor samples and 1 matched normal sample. In order to match the tumor samples, we downloaded the genome expression data of normal samples from the Genotype-Tissue Expression (GTEx) database (https://www.gtexportal.org/). Considering ultraviolet radiation as a high-risk factor for melanomas [24], we selected samples that were not exposed to the sun, which included 273 suprapubic skin tissue samples. After merging the genome expression data, limma package was used for data normalization, and abnormally expressed genes were screened for in tumor and normal samples. Meanwhile, the immune-related signatures were obtained from the InnateDB database (https://www.innatedb.ca/), and the pheatmap package was used to identify intersections and the expression immune signature in abnormally expressed genes obtained above. After transferring the gene names into the Entrez ID, we investigated their biological functions by performing Gene Ontology (GO) and Kyoto Encyclopedia of Genes and Genomes (KEGG) analyses of differentially expressed immune genes in melanoma with clusterProfiler, org.Hs.eg.db, enrichplot, and ggplot2 packages. The significantly enriched terms are indicated by dot plots and circle plots (with the GOplot package). Clinical information, including age, gender, TNM stages, tumor grades, and follow-up data, was obtained with TCGAbiolinks package.

### 2.2. Construction of a Melanoma Risk Scoring Model

To investigate the role of these differentially expressed immune genes in prognosis prediction, we constructed a risk model to evaluate the importance of these hub genes. First, univariate Cox regression analysis was performed to identify prognostic signatures from the differentially expressed immune genes screened. To reduce the variables, significant signatures with *P* < 0.01 were selected by the least absolute shrinkage and selection operator (LASSO). Next, multivariate Cox analysis was performed, and the risk score was calculated as follows: risk score = *∑*(*β*_*i*_∗Exp_*i*_), where *β*_*i*_, the coefficients, represented the weight of the respective signature and Exp_*i*_ represented the expression level. Based on the results, we established a risk score for each patient, where the median risk score was considered the cutoff value, and patients were classified into high- and low-risk groups. Distribution of the vital status in the two groups was illustrated by curve and scatter plots using reshape2, ggplot2, scales, and cowplot packages, and the levels of differentially expressed immune signatures among the two groups were represented by a heatmap plot.

### 2.3. Evaluation of the Association between Risk Score and Clinical Variables

First, for survival prediction with the survival package, we evaluated the hazard ratio of the immune signatures and identified the hub immune signatures by *P*Filter = 0.05. Next, we generated a receiver operating characteristic (ROC) curve with the timeROC package for OS prediction in the different groups. Additionally, Kaplan-Meier analysis was performed and a log-rank test was used to assess the correlation between risk score and survival probability. Second, for assessing clinical features, because the melanoma was not classified based on pathological grade, we obtained the clinical data from TCGA database and performed univariate single and multivariate multiple independent factor analyses (by the Wilcoxon rank-sum test) to investigate the underlying relationships between risk score and clinical features, including age, gender, and TNM stage.

### 2.4. Evaluation of the Association between Risk Score and Immune Infiltration

From the TIMER database, we download the profiles that matched TCGA data to estimate the abundance of six immune infiltrates (B cells, CD4^+^ T cells, CD8^+^ T cells, neutrophils, macrophages, and dendritic cells) by pathological estimations. Based on TIMER, the correlation between risk score/immune signature expression and tumor immunological features was determined by calculating the Pearson correlation coefficient and the estimated *P* value, which indicated the association between risk score and immune infiltration.

### 2.5. Evaluation of the Association between Risk Score and Immune Cell Abundance

To determine the tumor microenvironment characteristics, besides TIMER, we utilized CIBERSORT to estimate the fractions of 22 immune cell types in each sample, which are represented as a box plot. Furthermore, the Wilcoxon rank-sum test was performed to evaluate the correlation between immune cell abundance and risk score. Finally, the density of immune cells in the two groups was illustrated with a heatmap, which represented the levels of immune-infiltrating cells in the groups.

### 2.6. Statistical Analysis

For comparison of categorical variables, the chi-square (*χ*^2^) test was performed. The Wilcoxon rank-sum test, a nonparametric statistical test, was mainly used for comparing two groups, and the Kruskal-Wallis test was used for comparison of more than two groups. Differential gene expression data was analyzed by the “limma” package, which was also used for data normalization. Cox regression analysis or Kaplan-Meier analysis with log-rank test was performed using the “survival” package. All statistical analyses were performed using R software (version 3.6.1), and a *P* value < 0.05 was considered to be statistically significant.

## 3. Results

### 3.1. Identification of Prognostic Immune-Related Signatures in Melanoma

**Figure 1:**
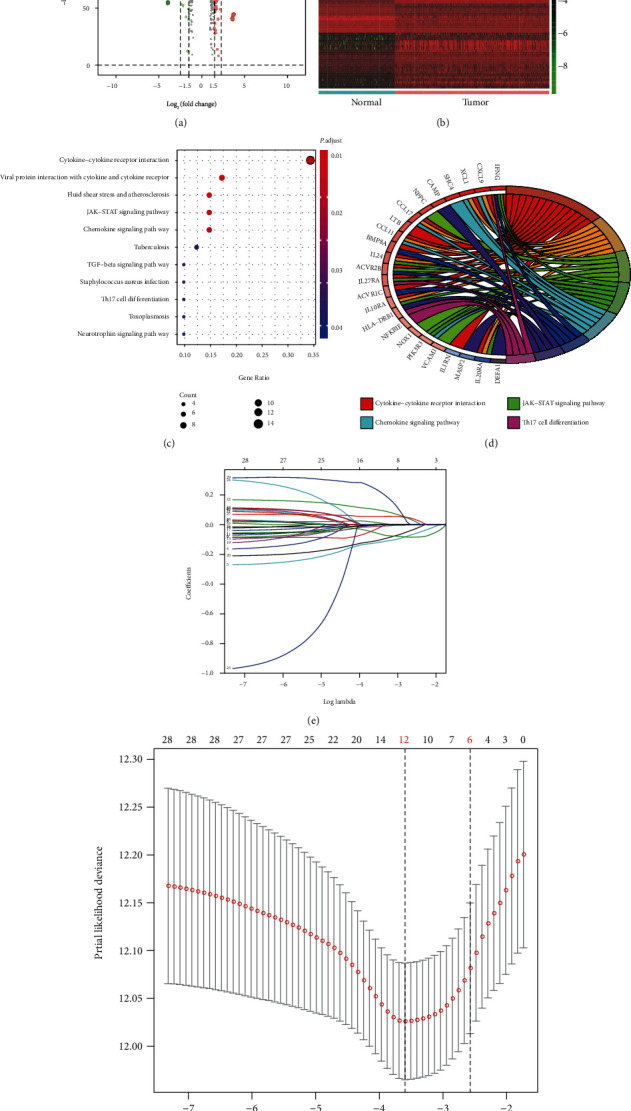
Identification of prognostic immune-related signatures in melanoma: (a) DEGs in melanoma vs. normal samples; (b) the intersection and differential expression of 91 immune-related signatures; (c, d) functional enrichment analysis revealed the potential immune-related crosstalks associated with prognostic immune signatures; (e, f) LASSO regression analysis was used to identify 12 hub tumor-associated immune signatures.

This study included 605 samples from TCGA and GTEx databases, including 471 tumor samples and 234 corresponding normal samples (not exposed to the sun; suprapubic). Excluding patients with insufficient clinical data, the complete clinical information is presented in Table 1. For subsequent experiments, we screened 345 tumor samples, which matched clinical and transcriptome data from the databases. After data normalization with the limma package, 968 differentially expressed genes (DEGs) were identified from transcriptome profiles with ∣log fold change | >1 and false discovery rate (FDR) < 0.05. The up- or downregulated DEGs are shown in Figure 1(a). From the InnateDB database, we obtained a list of 2498 immune-related genes and intersected 91 differential immune-related signatures. The expression of these signatures is displayed as a heatmap (Figure 1(b)). Functional enrichment analysis indicated that these immune-related DEGs participated in several areas of immune-related crosstalk, including cytokine-cytokine receptor interactions, Janus kinase/signal transducers and activators of transcription (JAK-STAT) signaling pathway, chemokine signaling pathway, and Th17 cell differentiation pathway (Figures 1(c) and 1(d)).

### 3.2. Risk Score and Model Assessment

**Figure 2:**
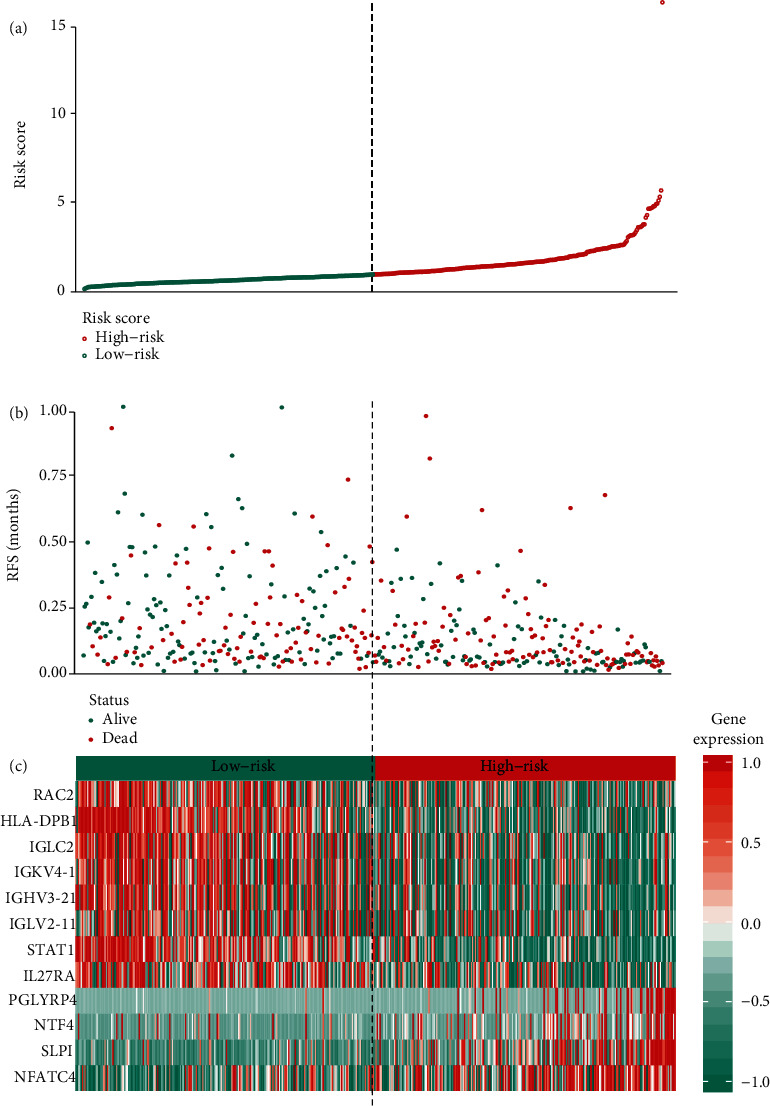
Establishment of a risk model: (a, b) vital status of patients based on the risk score; (c) the expression of identified hub immune signatures between low- and high-risk groups.

**Figure 3:**
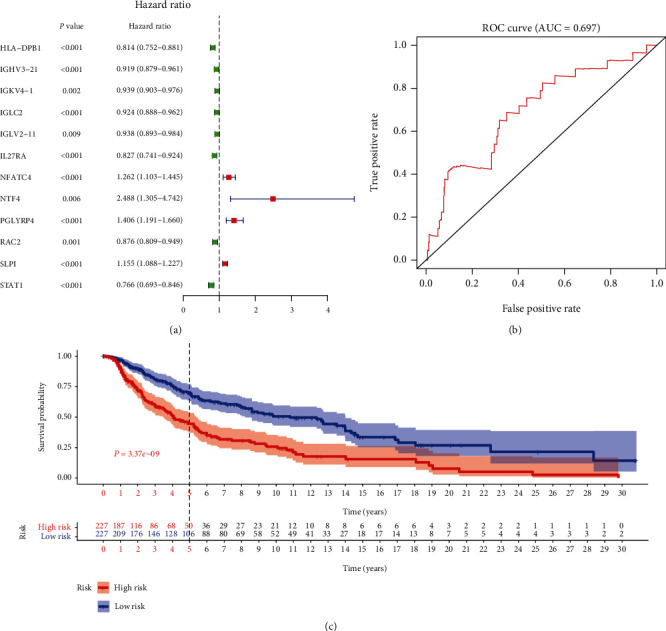
Validation of the risk score prediction model for melanoma prognosis. (a) Results of multivariate Cox analysis of 12 hub immune signatures are illustrated as a forest plot. (b) The 5-year AUC of ROC curve was 0.697, indicating high prediction accuracy. (c) Results of Kaplan-Meier analysis showed that patients in the high-risk group had poor survival outcomes (*P* < 0.001).

**Figure 4:**
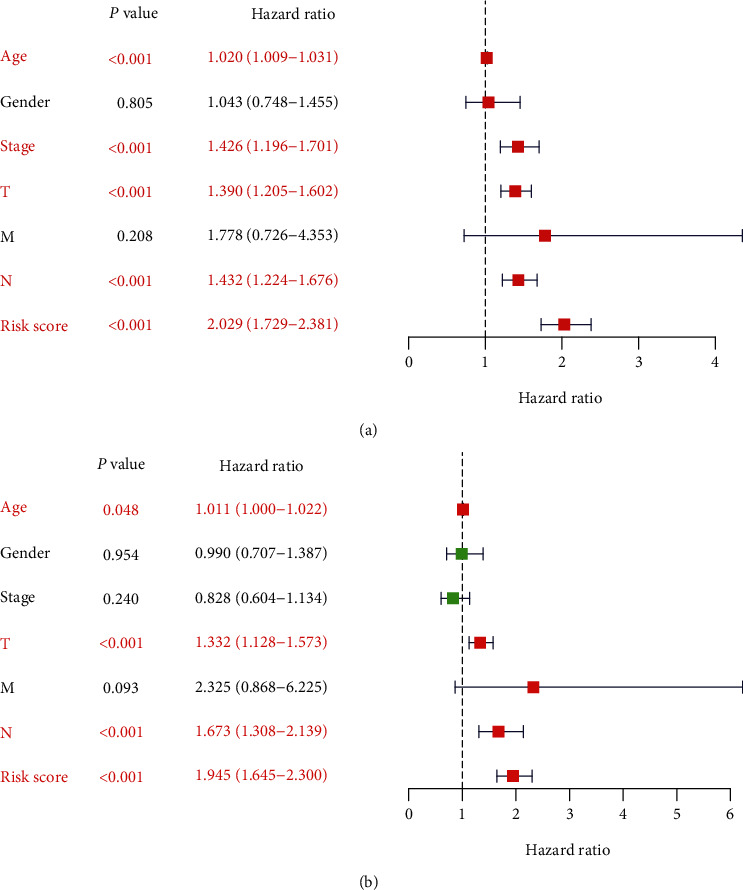
The correlation between risk score and AJCC-TNM stages. (a) The results of univariate prognostic analysis indicated that risk score was closely correlated to the stages, T and N. (b) The results of multivariate prognostic analysis indicated that risk score was closely associated with the T and N stages.

In order to identify prognostic genes among the selected immune-related signatures, Cox and LASSO regression analyses were performed for the establishment of a risk scoring model. The hub signatures were screened based on the OS results. First, the differential immune signature expression data were merged with the survival data, and 30 prognostic signatures with *P* < 0.01 were identified. These signatures were then evaluated by LASSO regression analysis (Figures 1(e) and 1(f)), and 12 hub immune genes were identified (Table 2). Finally, to define the weight of each gene, multivariate Cox regression analysis was performed to calculate the coefficient of these signatures. Based on these results, the risk score was calculated as follows: risk score = −HLA − DPB1∗0.219464 − IGHV3 − 21∗0.086658 + IGKV4 − 1∗0.080249 − IGLC2∗0.119770 + IGLV2 − 11∗0.120023 − IL27RA∗0.182126 + NFATC4∗0.180356 − NTF4∗0.932083 + PGLYRP4∗0.325587 + RAC2∗0.130861 + SLPI∗0.101763 − STAT1∗0.103435. The melanoma patients enrolled in this study were classified into low- and high-risk groups (227 patients/group). As shown in Figures 2(a) and 2(b), the low-risk group exhibited a lower survival risk. The hub gene expression data of the two groups are shown in Figure 2(c). The hazard ratio of each hub gene is shown in Figure 3(a), and the ROC plot generated is shown in Figure 3(b). The 5-year area under curve (AUC) was 0.697, which confirmed the prediction accuracy of the risk score for melanoma prognosis. The OS results are shown in Figure 3(c). As demonstrated by the Kaplan-Meier analysis, the high-risk group was associated with a poorer OS (*P* < 0.001) compared with the low-risk group. The correlation between risk score and AJCC-TNM stage is shown in Figure 4. The results of univariate prognostic analysis (Figure 4(a)) indicated that risk score is closely correlated to the stages, T and N (*P* < 0.001), and the results of multivariate prognostic analysis (Figure 4(b)) also indicated that risk score is closely correlated to the T and N stages (*P* < 0.01), suggesting the clinical significance of risk score.

### 3.3. Association between Immune Signatures and Tumor-Infiltrating Immune Cells

**Figure 5:**
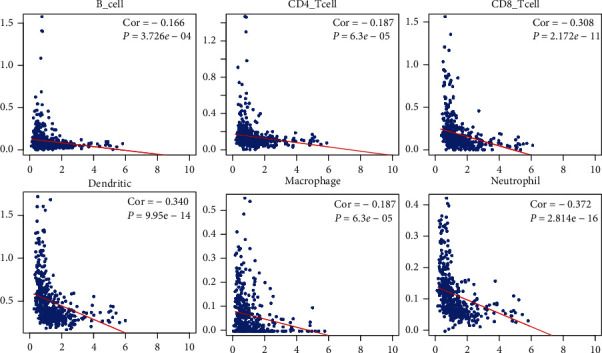
The relationship between 12 hub immune signatures/risk score and 6 tumor-infiltrating immune cells was evaluated using the TIMER database.

**Figure 6:**
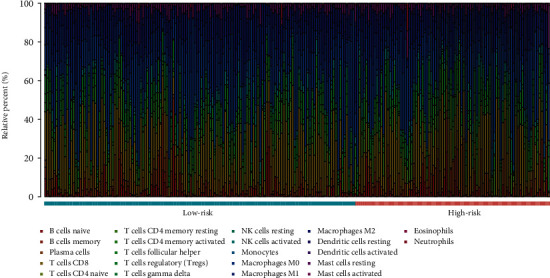
Estimation of fractions of immune cells using the CIBERSORT algorithm. The 22 immune cells were annotated by various colors, indicated below the legend.

In order to evaluate the role of infiltrating immune cells in the tumor microenvironment, we explored the function of immune signatures in immune infiltrates. First, we evaluated the relationship between 12 hub immune signatures/risk score and 6 immune cell types according to the TIMER database. As shown in Figure 5, risk score was closely associated with the immune cells. In addition, similar to risk score, all hub immune signatures were also associated with the immune cells (see Figure S1). Next, CIBERSORT algorithm was used to further estimate the fractions of 22 immune cells in each sample, after excluding samples with a calculated *P* value > 0.05. The sum of the immune fractions in each sample was equal to one. The various immune cells in each sample are illustrated as a box plot. (Figure 6).

### 3.4. Association between Risk Score and Tumor-Infiltrating Immune Cells

**Figure 7:**
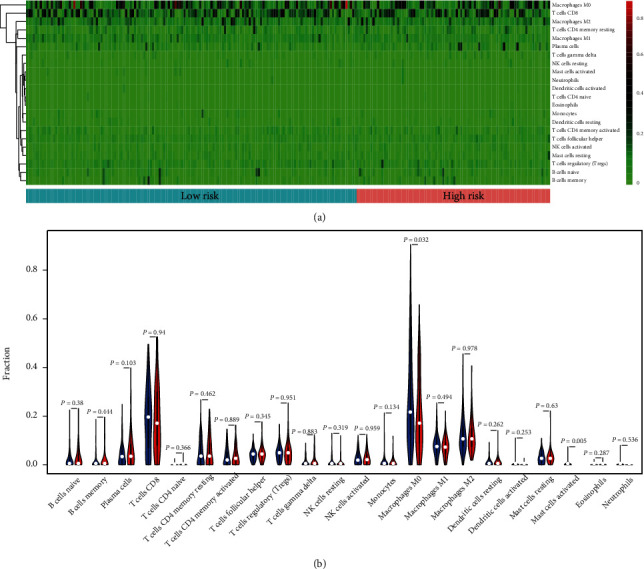
Differential distribution of immune cells among the two groups. (a) Heatmap illustrating the differences in infiltrating immune cells among the two groups. The colors ranging from green to red represent the infiltration density from low to high. (b) Wilcoxon rank-sum test was used to accurately compare the differences, and results showed that M0 macrophages (*P* = 0.032) and activated mast cells (*P* = 0.005) in the high risk-group displayed a significantly lower infiltration density.

As shown in Figure 5, we analyzed the potential association between risk score and distribution of tumor-infiltrating immune cells. First, the differences in infiltrating immune cells among the two groups were shown as a heatmap (Figure 7(a)). The low-risk group displayed a larger number of M0 macrophages (red color represents the infiltration density) compared with the high-risk group. Hence, M0 macrophages were regarded as the dominant immune cells. Next, the Wilcoxon rank-sum test was performed to evaluate the infiltration rate in the two groups. Results showed that M0 macrophages (*P* = 0.032) and activated mast cells (*P* = 0.005) in the high-risk group exhibited a significantly lower infiltration density. Based on these results, we inferred that risk factor was associated with lower immune infiltrates, resulting in poor survival outcomes.

## 4. Discussion

Previously, due to its lower incidence and mortality rate (compared with other cancer types), melanoma-related researches were focused on clinical treatment strategies rather than the underlying mechanisms. However, according to data from the SEER database (https://seer.cancer.gov/statfacts/html/melan.html), the incidence of melanoma has over tripled between 1975 and 2019. Although the annual mortality rate of melanoma was just 2.2% in 2019, MM has an MS of 8~12 months after metastasis. A systematic study of the immune microenvironment can provide a broader perspective on the MM immune infiltration signatures and thus reveal novel targets for immunotherapy. In the present study, using the TCGA-CTEe datasets, we established an immune-related risk signature for MM and constructed a risk model based on the results of univariate Cox regression and LASSO analyses. These signatures could predict the patients' OS, which was closely correlated to the clinical symptoms. According to the KEGG and GO analysis results, the selected signatures participated in cytokine-cytokine receptor interaction, Th1 and Th2 cell differentiation, and JAK-STAT signaling pathway. Moreover, a significant correlation between risk score and MM immune microenvironment was observed. A higher risk score indicated a lower density of infiltrating immune cells, a high immune risk microenvironment, deterioration of MM, and a poorer OS. The present study linked the immune-related signature ⟶ immune microenvironment ⟶ AJCC-TNM stages ⟶ patients' OS and revealed the effect of immune microenvironment on MM progression. The risk model established in this study can also be regarded as a predictor of the development, recurrence, and survival outcome of MM. Additionally, according to the results of multivariate Cox regression analysis, the 12 hub immune-related signatures screened may not only serve as independent predictors of MM in the clinic but also as potential immunotherapy targets for MM patients in the future.

Here, we identified 12 hub immune-related signatures, among which human leukocyte antigen (HLA), interleukin-27 (IL-27), nuclear factor of activated T cells, cytoplasmic 4 (NFATc4), and neurotrophin-4 (NTF4) were the most significant factors. HLA participates in the immune system, which induces immune responses via the antigen, thus playing a crucial role in immunological surveillance. As a member of the HLA family, HLA-II, a heterodimer of *α* (DPA) and *β* (DPB) chain, localizes in the cell membrane and is expressed in antigen-presenting cells, such as B lymphocytes, dendritic cells, and macrophages. HLA-DPB1 is derived from the *β* chain of HLA-II. In a previous research, Dhall et al. [25] showed that HLA-DPB1 functioned in presenting abnormal antigens in MM, thus stimulating melanoma progression and metastasis. In gene polymorphism research, HLA-DPB1 mutations have been regarded as signatures associated with gastric and lung cancers [26].

Compared with HLA-DPB1, IL-27 is another important signature specific for cutaneous cancer. In a previous research conducted by Dibra et al. [27], IL-27 was shown to play an unrecognizable role in promoting papilloma formation, which disrupted epithelial stem cell homeostasis/maintenance. A follow-up study demonstrated that [28], in a K15-KRASG12D mouse model, IL-27 accelerated the accumulation of endothelin A receptor-positive CD11b cells, a novel category of protumor inflammatory cells, thus establishing a premalignant niche and expanding the mutation of stem cells. In patients with squamous cell carcinoma, the distribution of IL-27 receptor subunit alpha-positive cells in the stroma was shown to be closely correlated to tumor dedifferentiation.

Many transcription factor families participate in the regulation of neurite growth, including the nuclear factor of activated T cell (NFAT) family [29]. NFATc4 belongs to the NFAT family and is mainly expressed in the nervous system, where it regulates neural functions, such as hippocampal plasticity, axon growth, neuron survival, and apoptosis in the brain [30]. In addition, NFAT family members could activate the immune response of T cells, as reported by Hessmann et al. [31]. In acinar cell plasticity and pancreatic cancer initiation, NFATc4 was found to be overexpressed and localized in the nucleus, thus activating the inflammation-induced epidermal growth factor receptor signaling pathway and upregulating the expression of Sox9. Therefore, we suggest that NFATc4 serves as a bridge between neuroscience and immune-oncology.

Similar to NFATc4, NTF4 is also expressed in the nervous system. However, there is limited research on its role in cancer. As an optic nerve disease signature, NTF4 has been commonly used for the assessment and identification of glaucoma [32]. A previous study by Shen et al. [33] showed that, after induction with specific receptor cleavages, NTF4 could suppress IL-6 family receptors and the Notch signaling pathway, which modulate protein kinase B (PKB/Akt) activity, thus decreasing the phosphorylation of STAT3. A previous study indicated that [34, 35] PKB/Akt signaling has been implicated in MM metastasis to distant organs, especially the brain. These findings suggest that NTF4 can modulate MM metastasis via the PKB/Akt signaling pathway.

The RAC protein family, belonging to the Rho GTPase superfamily, has three subtypes: Rac1, Rac2, and Rac3 [36]. The RAC protein family participates in the regulation of monocyte chemotaxis, stimulation of nicotinamide adenine dinucleotide phosphate (NADPH) oxidase activity, and generation of reactive oxygen species (ROS) [37, 38]. RAC1 was identified as the third most common recurrent mutation in melanomas and can be found in 4–7% of all patients [39]. It can be activated during deadhesion, contributing to superoxide production and RAS activation [40]. RAC2 targets NADPH oxidase, thus promoting ROS generation. In a previous study, Diebold and Bokoch [41] showed that RAC2 regulated electron transfer from NADPH to flavin adenine dinucleotide, thus controlling the inflammatory response of phagocytes downstream. Moreover, studies have shown that, during diallyl disulfide-induced apoptosis of human leukemia HL-60 cells, RAC2 is overexpressed [42]. With regard to its immunologic function, downregulation of RAC2 in MM skin tissues suggested that, similar to RAC1, RAC2 is widely involved in MM invasion and metastasis.

Secretory leukocyte protease inhibitor (SLPI), which serves as an important protective component of the mucosa and skin [43, 44], is an effective inhibitor of neutrophil elastase [45]. Because of the protease inhibitor site in C-terminal domains, SLPI can inhibit the activation of various serine proteinases, including chymotrypsin, trypsin, trypsin elastase, histoproteinase G, and mast cell chymotrypsin. Besides its anti-inflammatory [46] and prowound healing [47] effects, SLPI also acts as a modulator of innate immune responses of macrophages [48], which induce degradation of inhibitor of nuclear factor kappa-B (NF-*κ*B), thus leading to NF-*κ*B activation [49]. Overexpression of SLPI has been associated with the metastasis of breast cancer [50], gastric cancer [51], and malignant glioma [52], wherein it degrades the basement membrane and promotes the aggressiveness of these tumor types. In melanoma progression, SLPI is functionally related to kallikreins [53], which is an important signature in intercellular adhesion, keratinocyte differentiation, and cell exfoliation.

Localized in the cytoplasm, STATs can translocate into the nucleus and induce specific DNA binding after activation. The STAT family has dual functions in signal transduction and transcription regulation. In IL-2 therapy for melanoma, the absence of STAT1 was correlated with increasing clinical stage [54]. Further, via the eIF4F-STAT1-PD-L1 axis [55], STAT1 could inhibit proliferation and induce apoptosis, thus blocking the growth of melanoma cells [56]. However, some studies have indicated that STAT1 participates in late-stage melanoma progression [57]. Regarding its immunologic function, we suggest that STAT1 is widely involved in melanoma immune responses in a complex way.

In the present study, various immune signatures were found to be closely associated with melanoma prognosis as well as immune cell distribution. Furthermore, we investigated the correlation between immune cells and risk score. Results showed that, in the high-risk group, M0 macrophages (*P* = 0.023) and activated mast cells (*P* = 0.005) had a significantly lower infiltration density.

Although insignificant differences were found among other immune cells and M0 macrophages were the dominant immune cells (80.23 ± 0.22% in the high-risk group and 60.23 ± 2.61% in the low-risk group), it can be inferred that the decrease of immune-infiltrating cells in the tumor microenvironment of the high-risk group may be related to poor MM prognosis. Nevertheless, further investigations are needed to clarify the characteristics of other immune cells in the tumor microenvironment.

Taken together, we identified and validated 12 immune-related genes in melanoma based on immune infiltration and constructed a robust risk scoring model using these immune signatures. This risk model could accurately predict the prognosis of MM patients and the immune infiltration intensity in the MM microenvironment. The selected immune signatures may also serve as novel immune targets for MM immune-related treatments in the future.

Considering the low incidence and poor prognosis of melanoma, collection of clinical samples is difficult. In this study, we used a large number of cohort samples from TCGA and GTEx databases to investigate the expression of immune-related genes. Through analysis of immune infiltration, the regulation of immune-related genes in melanoma and their relationship with prognosis were studied in detail. Simultaneously, CIBERSORT and TIMER were used to analyze immune cells in the samples, which greatly improved research efficiency. Considering that this study was mainly carried out by bioinformatics, its clinical accuracy needs further validation. Moreover, the function of the identified biomarkers needs to be verified by PCR and western blotting experiments to determine its clinical value, which may provide a theoretical and therapeutic basis for the treatment of melanoma.

Due to the low incidence and poor prognosis of melanoma, collection of clinical samples is difficult. In our study, we analyzed clinical samples from TCGA and GTEx databases and evaluated the expression of immune-related genes in immune cells. Through analysis of immune cell infiltration, we identified a correlation between these immune-related genes and MM morbidity and prognosis. Furthermore, the use of CIBERSORT and TIMER improved the research efficiency of immune cell distribution in the samples.

Using the approach of this study, we can use existing research to systematically study immune inflammation without investigating underlying pathways or understanding the cell composition of the samples. This enables us to comprehensively analyze the immune and inflammatory patterns of melanoma reported in previous studies, evaluate the specific distribution of immune cells and the changes in corresponding biomarkers, and identify the biological relationship between them. Hence, our findings reveal a series of novel targets for the clinical treatment of MM. In addition, the feasibility of this study is high, thus providing a feasible method for future research on melanoma and other cancer types.

## Figures and Tables

**Table 1:** Baseline characteristics of 470 melanoma patients included in this study.

Variables	Count	Percentage (%)
Age (mean ± SD)	58.22 ± 15.73	
Follow-up (y)	4.96 ± 5.28	
Status		
Alive	259	(55.11)
Dead	211	(44.89)
Gender		
Male	290	(67.70)
Female	180	(38.30)
Pathological stage		
I/II NOS	14	(2.98)
Stage 0	8	(1.70)
Stage I	76	(16.1)
Stage II	140	(29.79)
Stage III	171	(36.38)
Stage IV	23	(4.894)
Unknown	38	(8.09)
AJCC-T		
T0	23	(4.89)
T1	42	(8.94)
T2	78	(16.60)
T3	90	(19.15)
T4	153	(32.55)
Tis	8	(1.70)
TX	47	(10.00)
Unknown	29	(6.17)
AJCC-N		
N0	235	(50.00)
N1	74	(15.74)
N2	49	(10.43)
N3	55	(11.70)
NX	36	(7.66)
Unknown	21	(4.47)
AJCC-M		
M0	418	(88.94)
M1	24	(5.11)
Unknown	28	(5.96)
Grade		
G1	—	—
G2	—	—
G3	—	—
G4	—	—
Unknown	1053	(100)
Risk score		
Low	227	(48.30)
High	227	(48.30)
Unknown	16	(3.40)

Abbreviations: TCGA: The Cancer Genome Atlas; AJCC: American Joint Committee on Cancer; TAIG: tumor-associated immune genes.

**Table 2:** Identification of 12 hub prognostic immune signatures based on a multivariate regression method.

Gene symbol	Description	coef	HR	HR.95L	HR.95H	*P*
HLA-DPB1	HLA class II histocompatibility antigen, DP beta 1 chain	-0.219	0.803	0.693	0.930	0.003
IGHV3-21	Immunoglobulin heavy variable 3-21	-0.087	0.917	0.819	1.026	0.131
IGKV4-1	Immunoglobulin kappa variable 4-1	0.080	1.084	0.977	1.202	0.129
IGLC2	Immunoglobulin lambda constant 2	-0.120	0.887	0.795	0.990	0.033
IGLV2-11	Immunoglobulin lambda variable 2-11	0.120	1.128	1.009	1.260	0.034
IL27RA	Interleukin-27 receptor subunit alpha	-0.182	0.833	0.739	0.940	0.003
NFATC4	Nuclear factor of activated T cells, cytoplasmic 4	0.180	1.198	1.036	1.384	0.015
NTF4	Neurotrophin-4	-0.932	0.394	0.118	1.309	0.128
PGLYRP4	Peptidoglycan recognition protein 4	0.326	1.385	0.996	1.925	0.053
RAC2	Ras-related protein Rac2	0.131	1.140	0.998	1.301	0.053
SLPI	Antileukoproteinase	0.102	1.107	1.007	1.217	0.035
STAT1	Signal transducer and activator of transcription 1-alpha/beta	-0.103	0.902	0.787	1.033	0.135

## Data Availability

The original data used to support the findings of this study was obtained from public databases (TCGA and GTEx).

## Supplementary Materials

Supplementary Materials Figure S1: correlation between tumor-infiltrating immune cells where pink represented the positive correlation while blue represented the negative correlation.
