# Supplementary figures and images for: Exploration of the Immune-Related Signatures and Immune Infiltration Analysis in Melanoma
Source: Anal Cell Pathol (Amst). 2021 Jan 16;2021:4743971. doi: 10.1155/2021/4743971 (PMC7826228; doi:10.1155/2021/4743971)

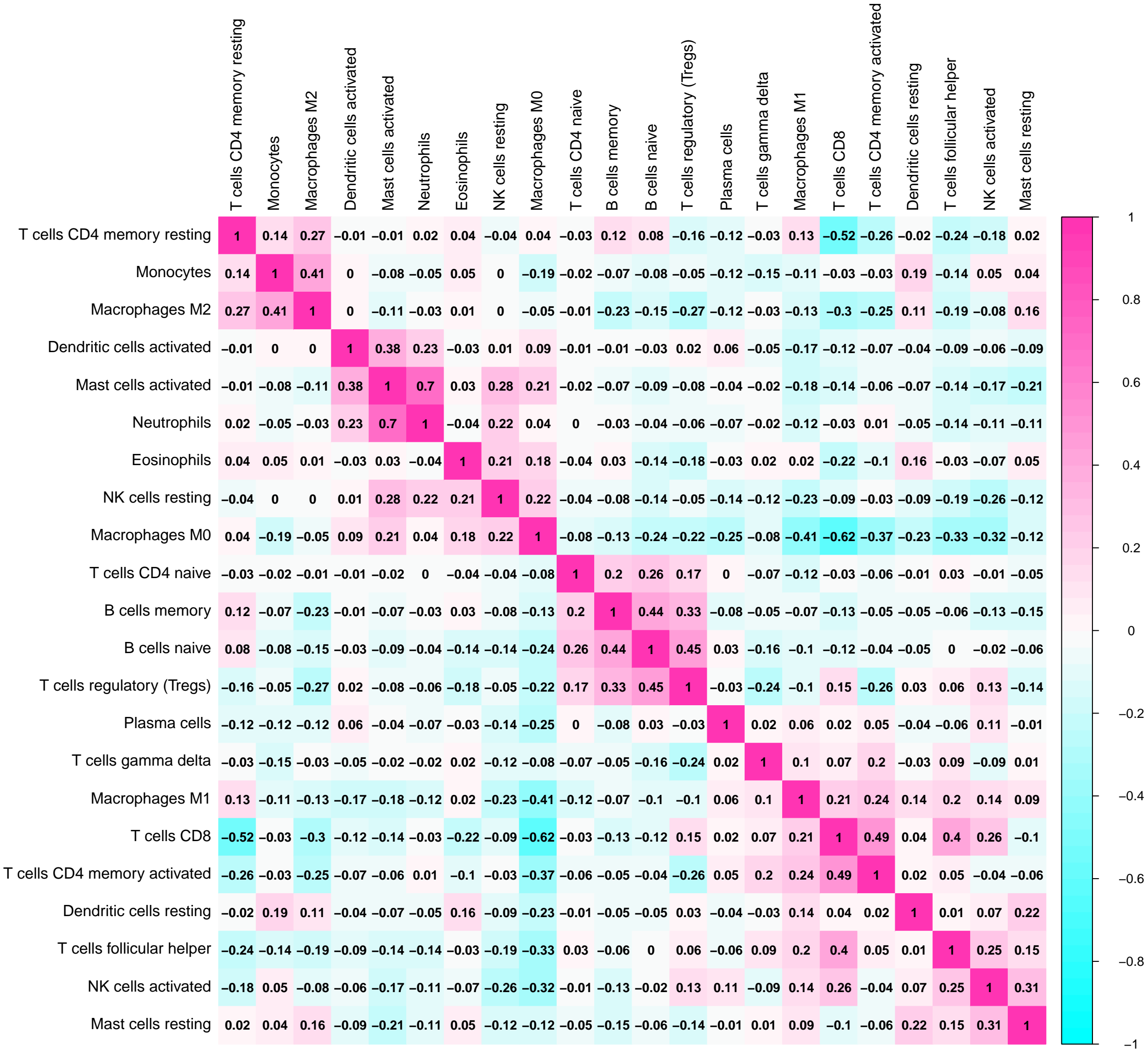

Supplement: Supplementary Materials — Figure S1: correlation between tumor-infiltrating immune cells where pink represented the positive correlation while blue represented the negative correlation. [file 4743971.f1.pdf]
